# Pharmacological isolation of postsynaptic currents mediated by NR2A- and NR2B-containing NMDA receptors in the anterior cingulate cortex

**DOI:** 10.1186/1744-8069-3-11

**Published:** 2007-04-30

**Authors:** Long-Jun Wu, Hui Xu, Ming Ren, Xiaoyan Cao, Min Zhuo

**Affiliations:** 1 Department of Physiology, Faculty of Medicine, University of Toronto, 1 King's College Circle, Medical Science Building, Room 3342, Toronto, Canada

## Abstract

NMDA receptors (NMDARs) are involved in excitatory synaptic transmission and plasticity associated with a variety of brain functions, from memory formation to chronic pain. Subunit-selective antagonists for NMDARs provide powerful tools to dissect NMDAR functions in neuronal activities. Recently developed antagonist for NR2A-containing receptors, NVP-AAM007, triggered debates on its selectivity and involvement of the NMDAR subunits in bi-directional synaptic plasticity. Here, we re-examined the pharmacological properties of NMDARs in the anterior cingulate cortex (ACC) using NVP-AAM007 as well as ifenprodil, a selective antagonist for NR2B-containing NMDARs. By alternating sequence of drug application and examining different concentrations of NVP-AAM007, we found that the presence of NVP-AAM007 did not significantly affect the effect of ifenprodil on NMDAR-mediated EPSCs. These results suggest that NVP-AAM007 shows great preference for NR2A subunit and could be used as a selective antagonist for NR2A-containing NMDARs in the ACC.

## Background

NMDA receptors (NMDARs) have pivotal roles in excitatory synaptic transmission and plasticity, and in various brain processes from memory formation to chronic pain [1,2]. NMDARs are tetrameric complexes, which contain two NR1 and two NR2 subunits (NR2A-D). The type of NR2 subunits determines not only gating properties but also signaling pathways of NMDARs [3,4]. Therefore, different subunit compositions confer NMDARs distinct roles in the regulation of neuronal functions. In consistence with this notion, NMDARs could undergo subunit-specific regulations under physiological or pathological conditions. For example, NR2A subunit gradually replaces NR2B in most brain areas during postnatal development [5], while NR2B but not NR2A is up-regulated in the anterior cingulate cortex (ACC) after peripheral inflammation [6].

Considering the distinct roles of NMDARs, dissection of their subtype-selective functions will promote our understanding of molecular mechanisms underlying physiological and pathological processes, such as memory and pain. Although pharmacological tools are powerful, subtype-selective antagonists for NMDARs are not well developed [7]. Most selective antagonists are ifenprodil and its derivatives (e.g. Ro25-6981), which are more than 200-fold preference for NR1/NR2B than for NR1/NR2A [8,9]. A relatively selective NR1/NR2A antagonist, NVP-AAM077 (NVP) was developed recently and found to have more than 100-fold preferential blockade of NR1/NR2A vs NR1/NR2B [10]. Using these antagonists, recent studies have shown that NR2A-containg NMDARs are required for LTP, whereas NR2B NMDARs are required for LTD [11,12]. However, the concept of subtype-dependent LTP and LTD was questioned by other studies that reported the lack of NMDA subtype receptor selectivity for bi-directional synaptic plasticity [13-18]. Moreover, some of these studies also argued that NVP is not sufficient to discriminate between NR2A- and NR2B-containing NMDARs, with less than 10-fold selectivity [19,20].

NR2A and NR2B are highly expressed in the ACC, a forebrain area involved in emotion, memory and pain [21,22]. Our recent results indicate that both NR2A and NR2B are required for the induction of cingulate LTP and LTD [17,18]. Since previous debates of antagonist selectivity are based on results mostly obtained from hippocampal neurons and transfected cells, we wanted to re-examine the pharmacological properties of NMDARs with NVP and ifenprodil in the ACC. By testing antagonist effects with different application sequences and concentrations, we found that NVP at concentration of 0.4 μM and 0.1 μM is likely to be relatively selective for NR2A-containing NMDARs in ACC neurons.

## Materials and methods

All adult C57BL/6 mice were purchased from Charles River and were maintained on a 12 h light/dark cycle with food and water provided *ad libitum*. The Animal Studies Committee at the University of Toronto approved all experimental protocols. Coronal brain slices (300 μm) containing the ACC from six- to eight-week-old C57BL/6 male mice were prepared using standard methods [23]. Slices were transferred to a submerged recovery chamber with oxygenated (95 % O_2 _and 5 % CO_2_) artificial cerebrospinal fluid (ACSF) containing (in mM: 124 NaCl, 2.5 KCl, 2 CaCl_2_, 2 MgSO_4_, 25 NaHCO_3_, 1 NaH_2_PO_4_, 10 glucose) at room temperature for at least 1 h.

Experiments were performed in a recording chamber on the stage of an Olympus BX51WI microscope (Tokyo, Japan) with infrared DIC optics for visualization of whole-cell patch clamp recording. Excitatory postsynaptic currents (EPSCs) were recorded from pyramidal neurons in layer II/III of the ACC with an Axon 200B amplifier (Molecular devices, CA) and the stimulations were delivered by a bipolar tungsten stimulating electrode placed in layer V. The recording pipettes (3–5 MΩ) were filled with the solution containing (mM): 145 CsMeSO_3_, 5 NaCl, 1 MgCl_2_, 0.2 EGTA, 10 HEPES, 2 Mg-ATP, 10 phosphocreatine, 0.1 Na_3_-GTP, 5 QX-314 (adjusted to pH 7.2 with CsOH). NMDA receptor-mediated EPSCs (NMDA EPSCs) were pharmacologically isolated in ACSF containing CNQX (20 μM), and picrotoxin (100 μM). Neurons were voltage clamped at -30 mV and NMDA EPSCs were evoked at 0.05 Hz. To study NVP- or ifenprodil-sensitive component in total NMDA EPSCs, NVP or ifenprodil was bath-applied for 10 min after obtaining stable baseline for 5 min. The NVP- or ifenprodil-sensitive component was calculated as the reduction of current in the corresponding drugs at the last one minute. NMDA EPSCs were fitted with single exponential function and decay time constant reflects the time decaying to 37% of NMDA EPSCs. Access resistance was 15–30 MΩ and was monitored throughout the experiment. All antagonists were applied through the perfusion solution. Data were discarded if access resistance changed more than 15% during an experiment. Results were analyzed by t-test to identify significant differences. All data are expressed as mean S.E.M. In all cases, *P *< 0.05 was considered statistically significant.

## Results and discussion

**Figure 1:**
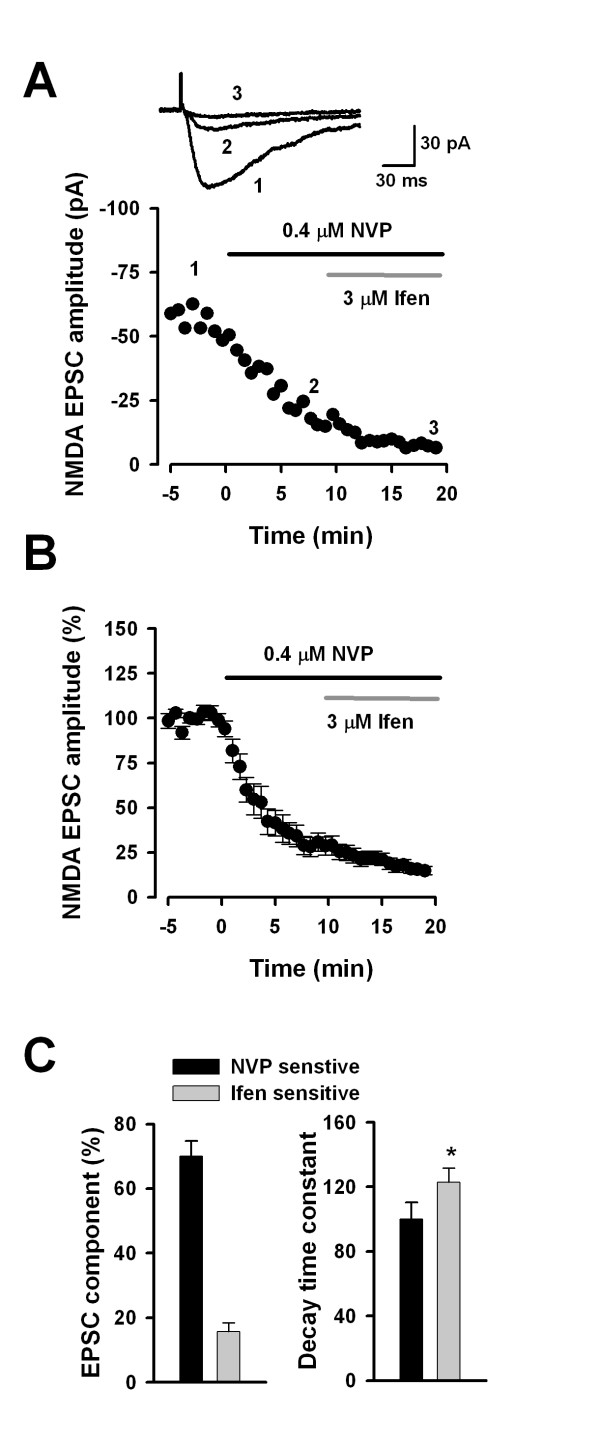
**Effects of NVP followed by ifenprodil on NMDA EPSCs in ACC neurons**. **A, **A single example showing the effect of sequential application of NVP (0.4 μM) and ifenprodil (3 μM) on NMDA EPSCs. The insets show average of 3 EPSCs at the time point (1, 2 and 3) indicated in the graph. **B, **Pooled data showing NVP largely decreased NMDA EPSCs and ifenprodil abolished the residual EPSCs. **C, **Bar graphs showing the average percentage (left) and decay time constant (right) of NVP- and ifenprodil-sensitive components after sequential application of NVP and ifenprodil.

Conventional whole-cell patch clamp recordings were performed in visually identified pyramidal neurons in layer II/III of ACC slices. NMDA EPSCs were isolated by adding CNQX (20 μM), an AMPA/KA receptor antagonist, and picrotoxin (100 μM), a GABA_A _receptor antagonist. Since ifenprodil is well accepted for its selectivity on NR2B NMDARs, whereas NVP on NR2A is a debate, we therefore employed the occlusion strategy to test NR2B component with or without pretreatment with NVP. If NVP is non-selective, that is, inhibiting NR2B, we then expected that the ifenprodil-sensitive component will be significantly smaller in the presence of NVP. To test this idea, we first applied NVP (0.4 μM) for 10 minutes and then applied ifenprodil (3 μM) for another 10 minutes (Figure 1A and 1B). We calculated the percentage of ifenprodil and NVP inhibition on NMDA EPSCs. The application of NVP decreased 70.0 ± 4.7 % (n = 9) while the following application of ifenprodil decreased 15.7 ± 2.7 % (n = 9) of total NMDA EPSCs (Figure 1C). This result is similar to our previous reports using NVP and Ro25-6981 [6,18].

We also examined the decay time constant of ifenprodil-sensitive and NVP-sensitive NMDA EPSCs (Figure 1C). Consistent with our previous reports [6,18], ifenprodil-sensitive component showed typical slower kinetics (τ = 122.7 ± 8.9 ms) compared with NVP-sensitive NMDA EPSCs (τ = 100.0 ± 10.5 ms, P < 0.05, paired t-test), showing they are preferentially mediated by NR2B- and NR2A containing NMDARs, respectively.

**Figure 2:**
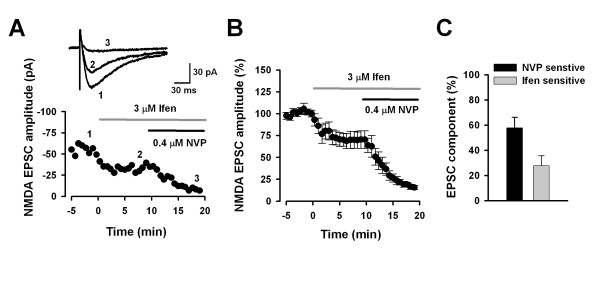
**Effects of ifenprodil followed by NVP on NMDA EPSCs in ACC neurons**. **A and B, **A representative example (**A**) and pooled data (**B**) showing the effect of sequential applications of ifenprodil (3 μM) and NVP (0.4 μM) on NMDA EPSCs. **C, **The bar graph showing the average percentage of NVP- and ifenprodil-sensitive components after sequential application ifenprodil and NVP.

Next, we alternated the sequence of drug applications, that is, we first applied ifenprodil and then applied NVP (Figure 2A and 2B). The application procedure revealed that ifenprodil (3 μM) reduced 27.6 ± 7.9 % (n = 8) of total NMDA EPSCs. The following perfusion of NVP (0.4 μM) further decrease 57.8 ± 8.3 % (n = 8) of total NMDA EPSCs (Figure 2C). We compared the percentage of ifenprodil-sensitive component between Figure 1C and Figure 2C, statistical results found no significant difference between two groups using different drug application sequences (P = 0.21, unpaired t-test). Similarly, we did not found the significant difference of NVP-sensitive component between two groups (P = 0.16, unpaired t-test).

We observed that NMDA EPSCs in different cells have various responses to ifenprodil and NVP. For example, in 17 cells tested, there are two cells showed around 50 % reduction when applied ifenprodil, whereas another 3 cells show less than 10 % of ifenprodil-sensitive component. To exclude the possibility that the insignificance is due to the variations among cells, we re-calculated the data by including the cells showing 10–50% ifenprodil-sensitive component in total NMDA EPSCs. No difference was found for ifenprodil-sensitive responses between the group treated with NVP followed by ifenprodil (18.6 ± 2.4 %, n = 7) and the group treated with ifenprodil followed by NVP (25.8 ± 2.2 %, n = 5, P = 0.08). Likewise, there is no difference in NVP-sensitive component between the two groups (65.4 ± 4.3 % vs 60.2 ± 3.9 %, P = 0.45). Taken together, these results suggest that NVP does not significantly affect NR2B component and could be used as a selective antagonist for NR2A-containing NMDARs in the ACC.

**Figure 3:**
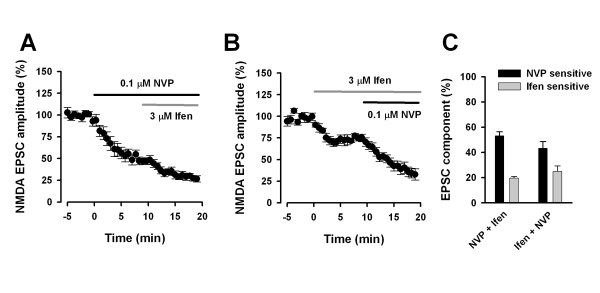
**Effects of the lower concentration of NVP and ifenprodil on NMDA EPSCs in ACC neurons**. **A, **Pooled data showing the effect of sequential applications of NVP (0.1 μM) and ifenprodil (3 μM) and on NMDA EPSCs. **B, **Pooled data showing the effect of sequential application of ifenprodil (3 μM) and NVP (0.1 μM) on NMDA EPSCs. **C, **Bar graphs showing the average percentage of NVP- and ifenprodil-sensitive component after sequential application of NVP (0.1 μM) + ifenprodil or ifenprodil + NVP.

To confirm the idea, we further tested the lower concentration (0.1 μM) of NVP (Figure 3). We found that the application of NVP (0.1 μM) decreased 53.2 ± 3.2 % (n = 7) while the following application of ifenprodil (3 μM) decreased 19.6 ± 1.3 % (n = 7) of total NMDA EPSCs (Figure 3A and 3C). Under these conditions, ifenprodil-sensitive component showed slower kinetics (τ = 123.5 ± 7.9 ms) compared with NVP-sensitive NMDA EPSCs (τ = 95.3 ± 8.2 ms, P < 0.05, paired t-test). When we alternated the sequence of drug application, the perfusion of ifenprodil decreased 24.9 ± 4.4 % (n = 5) and following NVP decreased 43.3 ± 5.3 % (n = 5) of total NMDA EPSCs (Figure 3B and 3C). No difference was found for percentage of ifenprodil-sensitive component between two groups (P = 0.27, unpaired t-test, Figure 3C). Similarly, no difference was found for percentage of NVP-sensitive component between two groups (P = 0.16, unpaired t-test, Figure 3C). Therefore, the lower concentration (0.1 μM) of NVP did not significantly affect NR2B-containing NMDARs in the ACC neurons.

**Figure 4:**
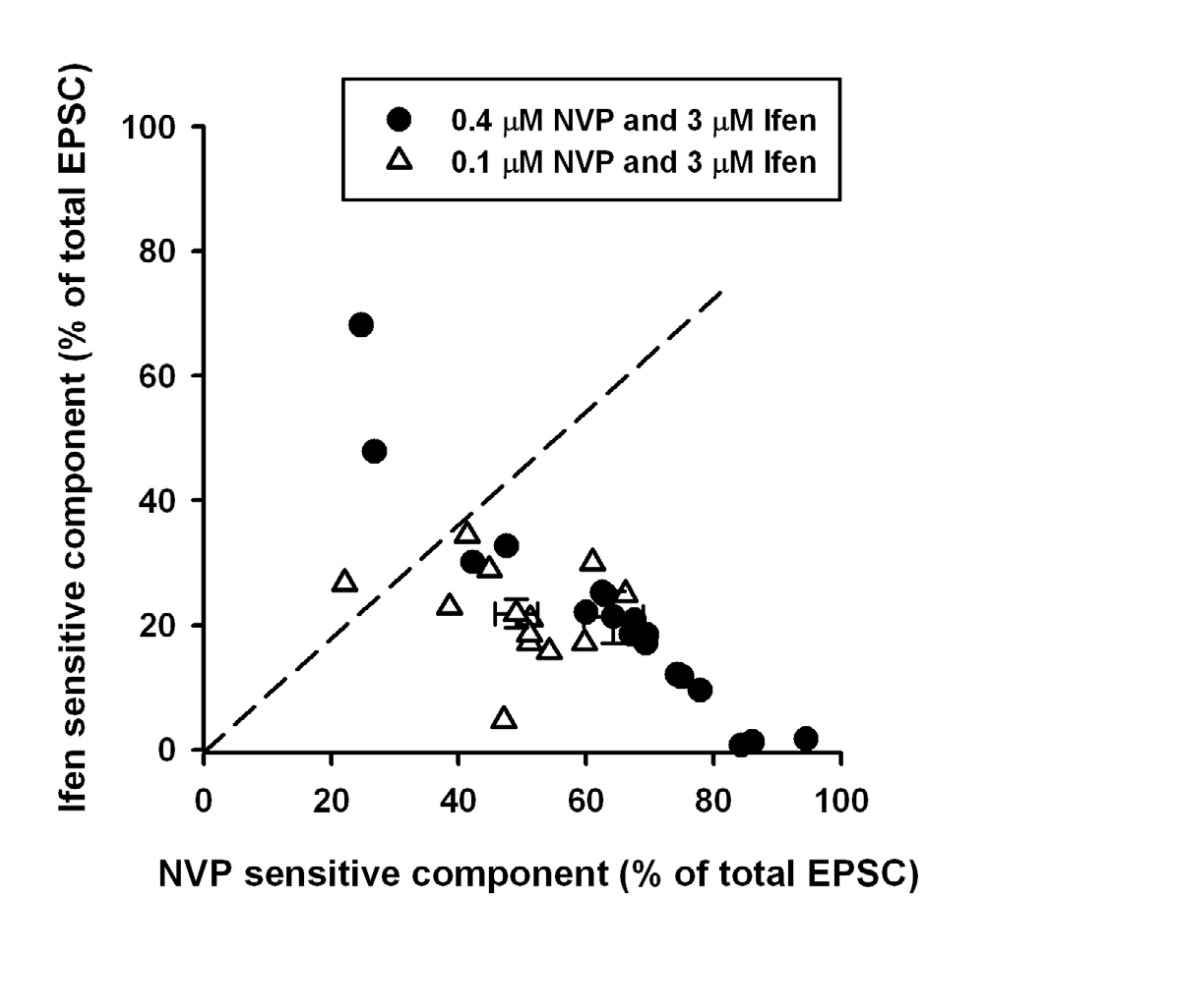
**Pooled data of NR2B and NR2A component in ACC neurons**. The percentages of NVP- and ifenprodil-sensitive components in each neuron were plotted in two groups: 0.4 μM NVP + 3 μM ifenprodil (filled circle) and 0.1 μM NVP + 3 μM ifenprodil (open triangle). Although no difference in ifenprodil-sensitive component, there is significant shift in NVP-sensitive component in two groups.

Since alternating sequences of drug application did not significantly affect their effects on NMDA EPSCs, the data were then pooled together (Figure 4). We found that NR2A component obtained by application of 0.4 μM NVP and 0.1 μM NVP is 64.3 ± 4.7 % (n = 17) and 49.0 ± 3.4 % (n = 12), which are significantly different from each other (P < 0.05, unpaired t-test). In contrast, there is no difference of NR2B component by 3 μM ifenprodil in two groups, which was 21.3 ± 4.2 % (n = 17) and 21.8 ± 2.3 % (n = 12) (P = 0.93, unpaired t-test), respectively. Therefore, high concentration of NVP (0.4 μM) did not affect NR2B component, whereas lower concentration of NVP (0.1 μM) is not effective enough in inhibiting NR2A-containing NMDA receptors in ACC neurons.

In the presence study, we used pharmacological tools to isolate postsynaptic currents mediated by NR2A- and NR2B-containing NMDARs, respectively. By alternating sequence of drug applications, we found that the presence of NVP did not significantly affect the effect of ifenprodil on NMDA EPSCs, suggesting that NVP is relatively selective for NR2A-containing NMDARs. There are several concerns related to our conclusions. First, it has been reported the regional difference of NMDARs in the ACC and hippocampus, such as NR2A/NR2B ratio, phosphorylated NR2A and NR2B [18] as well as regulations under chronic pain conditions [6]. Therefore, the current results may not fully applied to other brain areas including hippocampus. Indeed, it has been reported that 0.4 μM NVP could block one third of Ro-6981-sensitive NMDA EPSCs in cultured hippocampal neurons [24]. In the current study, due to the difficulty in washing out the drugs in slices, we cannot use the same way to calculate the possible effect of NVP on ifenprodil-sensitive component; Second, although it is difficult to reconcile the discrepancy for NVP and ifenprodil selectivity in different systems, the various reports may be due to complex subunit composition, spatial distributions of NMDARs, and their related scaffolding molecules [4,7]. For instance, triheteromeric NMDA receptor, NR1/NR2A/NR2B, has been found in native tissues, particularly in the cortex [5,25]. Moreover, it was shown that NR1/NR2A/NR2B is also highly sensitive to ifenprodil [26]. Alternatively, it has been proposed that there are potential interactions for NR2A and NR2B [27], which may also account for the variability. Therefore, first perfusion of NVP or ifenprodil might affect the following effect of ifenprodil or NVP. Third, due to the limitation of conventional whole-cell recording, we observed the rundown of NMDA EPSCs during 25 minutes in some of neurons (3 out 7 neurons). Although the pooled data showed no significance in the reduction of NMDA EPSCs (12.9 ± 6.6 % of control, n = 7, P = 0.13, paired t-test), the result may confound the contribution of NVP- and ifenprodil-sensitive component. Last, we were aware the variability of percentage of NR2B component in ACC neurons. In all tested 29 neurons, we found 4 neurons have much smaller ifenprodil-sensitive current (<10% of total currents, Figure 4). Future experiments (e.g. single cell RT-PCR) are needed to test the possibility that whether these neurons express less NR2B subunits.
